# Potential Infectious Etiology of Behçet's Disease

**DOI:** 10.1155/2012/595380

**Published:** 2011-12-29

**Authors:** Massimiliano Galeone, Roberta Colucci, Angelo Massimiliano D'Erme, Silvia Moretti, Torello Lotti

**Affiliations:** Department of Dermatological Sciences, University of Florence, Florence 50129, Italy

## Abstract

Behçet's disease is a multisystem inflammatory disorder characterized by recurrent oral aphthous ulcers, genital ulcers, uveitis, and skin lesions. The cause of Behçet's disease remains unknown, but epidemiologic findings suggest that an autoimmune process is triggered by an environmental agent in a genetically predisposed individual. An infectious agent could operate through molecular mimicry, and subsequently the disease could be perpetuated by an abnormal immune response to an autoantigen in the absence of ongoing infection. Potentia bacterial are *Saccharomyces cerevisiae*, mycobacteria, *Borrelia burgdorferi*, *Helicobacter pylori*, *Escherichia coli*, *Staphylococcus aureus*, and *Mycoplasma fermentans*, but the most commonly investigated microorganism is *Streptococcus sanguinis.* The relationship between streptococcal infections and Behçet's disease is suggested by clinical observations that an unhygienic oral condition is frequently noted in the oral cavity of Behçet's disease patients. Several viral agents, including herpes simplex virus-1, hepatitis C virus, parvovirus B19, cytomegalovirus, Epstein-Barr virus and varicella zoster virus, may also have some role.

## 1. Introduction

Behçet's disease is a multisystem inflammatory disorder characterized by recurrent oral aphthous ulcers, genital ulcers, uveitis, and skin lesions and generally presents with remissions and exacerbations. It can frequently involve the joints, gastrointestinal tract, and central nervous system [1, 2].

## 2. Epidemiology

Behçet's disease is most prevalent along the “Silk Road,” an ancient trading route between the Mediterranean and East Asia, where it is a major cause of morbidity. In Turkey, the country with the highest incidence of the disease, the prevalence is estimated to be between 110 and 420 per 100.000, whereas that in Japan is 13–20 per 100.000, and the prevalence in the UK and USA is estimated at 1-2 per 100.000. The typical age of onset is in the third or fourth decade of life and the male-to-female ratio varies with ethnic origin [1, 3, 4].

The study of migrant populations led to interesting epidemiological findings. Individuals from endemic areas who have immigrated to areas with low prevalence of the disease have an intermediate risk for developing the disease, which points that environment has some role in Behçet's disease. Turkish individuals who have emigrated to Germany have a significantly lower risk of disease than individuals of Turkish origin living in Turkey, although their risk remains higher than that of the native German population. Similarly, the disease is virtually unknown in Japanese immigrants to Hawaii, mainland USA, or South America despite a high prevalence in Japan [5, 6].

## 3. Pathogenesis

The cause of Behçet's disease remains unknown, but epidemiologic findings suggest that an autoimmune process is triggered by an infectious or environmental agent (possibly local to a geographic region) in a genetically predisposed individual [7, 8]. Whatever the stimulus is, the target tissue seems to be the small blood vessels, with various consequences of either vasculitis and/or thrombosis in many organ systems [9].

The genetic susceptibility is strongly associated with the presence of the HLA-B51 allele, with a stronger association in Turkish and Japanese patients in comparison to Caucasians. The unusual geographic distribution of Behçet's disease and its close association with HLA-B51 may be the strongest indicator that certain genes are directly responsible for Behçet's disease or of indirectly promoting the characteristics of the underlying inflammatory changes. Other genes located outside the HLA region have been also proposed, including genes of coagulation factor V, intercellular adhesion molecule-1 (ICAM-1), and endothelial nitric oxide synthetase [10–12].

As is the case of other autoimmune diseases, there is interest in an infectious etiology. Although there is no information supporting the role of a single microorganism as the specific cause, a problem with dysregulation in innate immunity, with an altered response to more than one infectious agent, is a generally accepted theory. An infectious agent could operate through molecular mimicry. This mimicked interaction or false signalling could attract the inflammatory cells into the field of action, and this may in turn result in vasculitis. Subsequently, the disease could be perpetuated by an abnormal immune response to an autoantigen in the absence of ongoing infection [13].

A viral cause was first postulated by Behçet in 1937 [14]. Evidence of ongoing infection with a variety of viral agents has been sought. However, often there is only a history of previous infection and/or seropositivity [2]. Although herpetiform ulcers are unusual, herpes simplex virus-1 (HSV-1) is currently the most common virus associated with Behçet's disease. HSV DNA and serum antibodies against the virus have been found in a higher proportion of patients with Behçet's disease than in controls, and circulating immune complexes with the HSV-1 antigen have been reported. HSV DNA has been demonstrated in the genital and intestinal ulcers, but not in oral ulcers. However, anti-HSV immunity is also common in normal subjects, and results about therapeutic effects of antiviral treatment in Behçet's disease are scarce and controversial [15, 16]. Several other viral agents, including hepatitis C virus, parvovirus B19, cytomegalovirus, Epstein-Barr virus, and varicella zoster virus, may also have some role [17–21].

Potential bacteria are a variety of streptococcal antigens, *Saccharomyces cerevisiae*, mycobacteria, *Borrelia burgdorferi*, *Helicobacter pylori*, *Escherichia coli*, *Staphylococcus aureus*, *Mycoplasma fermentans* [22–27]. The infectious model is also supported by observations that oral ulcers precede the establishment of disease by months or years and disease relapses are frequent. Thus, oral microbial flora have long been implicated in the pathogenesis. The most commonly investigated microorganism is *Streptococcus*. The relationship between streptococcal infections and Behçet's disease is suggested by clinical observations that an unhygienic oral condition including periodontitis, decayed teeth, and chronic tonsillitis is frequently noted in the oral cavity of Bechet's disease patients [28]. It is not clear that the predisposition of the patients is correlated with streptococcal infection, but the uncommon oral *Streptococcus sanguinis* serotypes (called KTH-1) and antibodies against the bacteria are significantly increased in the oral flora and serum, respectively, of patients with the disease compared with healthy controls [29]. The patients show strong delayed-type cutaneous hypersensitivity reactions against streptococcal antigens in skin tests and sometimes Behçet's disease symptoms were provoked by skin injection of the antigens [30]. The new criteria included hypersensitivity skin reactions against streptococci in the diagnosis as one of the references and the levels of disease severity of Behçet's disease patients [31]. *S. sanguinis* antigens share a sequence of amino acids with one of the protein classes of the cellular membranes called heat shock proteins (HSPs), which are expressed above the cellular membrane in response to physiological shocks and microbial stimulus [12]. Thus, HSPs are possible candidate antigen for Behçet's disease. Particularly, the peptides of 65 kDa (HSP-65) derived from the bacteria show considerable homology with those of the human 60 kDa (HSP-60). Moreover, mycobacterial and human HSPs have over 50% in sequence homology [32]. Studies have shown that *S. sanguis* and HSP 60/65 kDa activate *γδ*T cells in Behçet's disease patients but not controls. It is suggested that, following the bacterial stimulus, mucous cells express HSPs which are antigenic and reactive antimucous T cells in susceptible individuals (molecular mimicry model) [30]. As for the most other autoimmune disorders, the Th1-type polarization is predominant in Behçet's disease [7]. *γδ*T lymphocytes have a role in the immune response to infections and in autoimmunity by recognizing bacteria-derived and autologous antigens. Patients with Behçet's disease have increased numbers of activated *γδ*T cells (in circulation and in mucosal lesions), which produce inflammatory cytokines, including IFN-*γ*, TNF-*α*, and IL-8. Culture of *γδ*T lymphocytes from Behçet's disease patients proliferates in response to mycobacterial HSP-derived peptides and in response to products from microorganisms in oral ulcers [11, 33]. Complex interactions between T cells, antigen presenting cells, (APCs) and neutrophils are involved in the immune pathogenesis of Behçet's disease. Neutrophils are hyperactive in Behçet's disease, with increased chemotaxis, phagocytosis, superoxide production and myeloperoxidase expression and produce several cytokines [34]. Behçet's disease lesions might be induced with vascular reaction or lymphocytic vasculitis as the immunological reaction by the APCs expressing the *S. sanguinis* antigens [30]. Moreover, the amino acid sequence of the peptides of Bes-1, a gene derived from oral *S. sanguinis*, shows more than 60% similarity to the human intraocular ganglion peptide, Brn-3b. These results suggest that Bes-1 might be an inducer for the retinal and neural involvement possible in Behçet's disease patients [35].

Pustular skin lesions are often not sterile and may contain *Staphylococcus aureus* and *Prevotella *species. Whether these pustules are secondarily infected or whether the infections play a pathogenic role in the development of pustular lesions remains to be determined [36].

Anti-*S. cerevisiae* antibodies (ASCAs) may be especially common in intestinal Beçhet's disease and are also increased in healthy relatives of patients, according to the study by Choi et al. who evaluated whether ASCA expression is associated with clinical findings at diagnosis and the clinical course of intestinal Behçet's disease and found that the ASCA-positive rate was 44.3% in intestinal Behçet's disease but was not related to clinical findings at diagnosis and cumulative relapse rates [37].

Mycoplasmas are known to exhibit molecular mimicry to eukaryotic structures that may modulate immune responses [27, 38].

## 4. Therapeutic Implications

Antiseptic agents and antibiotics are used to control microbial contamination and secondary infection [39]. Chlorhexidine gel and triclosan have been shown to reduce the number, pain severity, and duration of aphthous ulcers [40, 41].

Antibiotics, especially tetracycline, has been widely used in oral ulcers of Behçet's disease for years. Tetracycline mouthwash decreases pain severity and duration of oral ulcers. Minocycline mouthwashes as compared to topical tetracycline rinses resulted in significantly improved pain control, by reducing the severity and duration of pain [42]. Minocycline reduces not only the growth of oral streptococci, but also suppresses interleukins production from T cells [43]. The effect of penicillin on mucocutaneous lesions and arthritis has been examined in two different studies, and it was found that prophylactic penicillin treatment reduced both the mucocutaneous lesions and the arthritis episodes. Other studies also showed that combination therapy, 1200000 units of benzathine-penicillin injected monthly plus 1 mg of oral colchicine daily for 4 months, was effective to suppress Behçet's disease symptoms, compared to colchicine monotherapy [44–46].

Acyclovir is not effective in the treatment of oral and genital ulceration [47].

## 5. Conclusion

The role of microorganisms in the pathogenesis of Behçet's disease has long been investigated, and there are ample data on several microorganisms. However, none of these infectious agents have been proved to cause Behçet's disease. Thus, a complex immune response might be generated to a group of microorganisms that share common antigens rather than to an infection due to a specific single microorganism.
